# Melatonin attenuates cardiac oxidative stress in diabetic rats following acute exhaustive exercise

**DOI:** 10.1016/j.cstres.2025.100126

**Published:** 2025-10-17

**Authors:** Carol Nguyen, Rafael Ishihara Figueiroa, Cristiano Mendes da Silva, Elaine Hatanaka, Gary Sweeney, Rafael Herling Lambertucci

**Affiliations:** 1 Department of Biology, York University, Toronto, Canada; 2 Department of Biosciences, Federal University of Sao Paulo (UNIFESP), Santos, Brazil; 3 Institute of Physical Activity and Sport Sciences, Cruzeiro do Sul University, Sao Paulo, Brazil

**Keywords:** Diabetic cardiomyopathy, Reactive species, Antioxidant, Diabetes, Cardiac tissue

## Abstract

**Introduction:**

Diabetes mellitus affects millions of people worldwide and there is evidence linking the increase of oxidative stress to the development of diabetic cardiomyopathy. Melatonin has been found to possess powerful antioxidant properties *via* modulating both enzymatic and non-enzymatic antioxidant systems.

**Objective:**

To evaluate the antioxidant potential of melatonin on the heart of diabetic animals at basal conditions and following 2 h of strenuous exercise.

**Methods:**

Diabetic animals were divided into two groups: non-supplemented and supplemented (melatonin). We evaluated oxidative stress biomarkers, total glutathione amount, oxidative stress index and antioxidant enzymes mRNA expression, immediately after an exhaustive exercise (IA group), and 2 h after exhausted exercise (2 h group). We also included a non-exercised group (0 h).

**Results:**

Comparing to non-exercised animals, exercise immediately induced an increase of nitrite and total antioxidant status in non-supplemented and supplemented animals, respectively. In melatonin-supplemented animals, the oxidative stress index decreased immediately after exercise (IA group) compared to non-exercised animals (0 h), an effect not seen in the non-supplemented group. Compared to non-supplemented, melatonin supplementation was shown to attenuate TBARS at all time points and increase total glutathione content at times 0 h and IA. mRNA expression of some antioxidant enzymes (CAT and GPX) was modulated by melatonin, especially when associated with exercise [catalase (CAT) and Cu, Zn superoxide dismutase (SOD)].

**Conclusion:**

Our findings demonstrate that melatonin confers antioxidant protection to the diabetic heart, primarily by increasing glutathione levels and attenuating lipid peroxidation. This establishes a protective state that enhances cardiac resilience, and the combination of melatonin and exercise may offer synergistic benefits against acute, stress-induced oxidative damage in diabetic animals.

## Introduction

Diabetes mellitus (DM) is a condition consisting of an inability to produce or properly utilize insulin, often resulting in a hyperglycemic status. This disease has become a growing pandemic, affecting millions of people worldwide.^1^ As of 2017, there were an estimated 425 million people around the world living with diabetes, according to the International Diabetes Federation. By 2045, the prevalence is expected to increase to 693 million people, not to mention the growing prevalence of both Type 1 and Type 2 diabetes in children under 18 years old especially due to unhealthy dietary choices as well as the adoption of sedentary lifestyles.^2^

Chronic complications of DM include micro- and macrovascular implications, from diabetic nephropathy, retinopathy, and neuropathy to increased risk of atherosclerosis, stroke, or cardiovascular disease.^3^, ^4^ However, one of the leading causes of death in diabetic patients is heart failure not attributed to coronary artery disease or hypertension, which is known as diabetic cardiomyopathy (DCM). Recent evidence has corroborated the contribution of mitochondrial dysfunction, namely the damages of oxidative stress, in the pathogenicity of DCM.^5^, ^6^ Oxidative stress resulting from hyperglycemia-induced reactive oxygen species production is of considerable interest as a major factor in the onset of this diabetic complication.^7^, ^8^

Healthy cells have both enzymatic and non-enzymatic antioxidant systems that provide some mechanisms of quenching excessive reactive oxygen species (ROS), resulting in a healthy balance of ROS production and elimination.^9^ Cellular enzymatic antioxidants include catalase (CAT), superoxide dismutase (SOD), and glutathione peroxidase (GPx). Non-enzymatic antioxidants include vitamins C and E as well as glutathione.^10^ Increased use of antioxidants has been investigated in therapy strategies to combat diabetic complications involving oxidative stress.^11^ Techniques that detect antioxidant responses can provide a useful foundation for examining the effect of potential therapeutic treatments, such as melatonin, in diabetic tissues damaged from oxidative stress.^12^

Melatonin, a hormone secreted from the pineal gland, has been found to possess antioxidant properties. It has been observed to combat oxidative stress directly as a ROS scavenger and indirectly through the melatonin-induced stimulation of antioxidant enzymes.^13^, ^14^ Melatonin has been shown to result in the up-regulation of SOD, CAT, and GPx mRNA expression in cells undergoing oxidative stress, either by H_2_O_2_ or UV radiation induction.^15^, ^16^ Moreover, the hormone has been used as an antioxidant therapeutic tool in recent studies wherein melatonin carried a cardioprotective effect against DCM^17^ as well as positive effects against oxidative stress in various diabetic tissues.^18^

Previous studies have already demonstrated the cardioprotective effects of melatonin in diabetes under basal conditions^17^, ^18^ and in response to chronic exercise training,^19^ however, it still remains to be fully elucidated how melatonin supplementation can modulate the immediate response of the cardiac antioxidant system to an acute and exhaustive physiological stressor. This distinction is critical, as it addresses the heart's resilience to a sudden challenge rather than its long-term adaptation. Furthermore, the dynamic, time-dependent nature of this potential protection has not been characterized yet. Therefore, in this study, we proposed to investigate the antioxidant potential of melatonin on the heart of diabetic animals not only at a basal condition but also at specific time points following a single, strenuous exercise session. We hypothesized that melatonin would protect the heart from oxidative stress by modulating the cardiac antioxidant system, consequently reducing biomarkers of oxidative damage.

## Methods

### Animals

All procedures involving animals were approved by the Ethical Committee of the Cruzeiro do Sul University (N° 002-2016 protocol), and the experiments were performed according to the Guidelines for the Care and Use of Laboratory Animals. Thirty-five-day old male Wistar rats (255 ± 22 g) were kept at constant temperature (21 °C) and standardized humidity (55%), under an inverted light/dark cycle (12/12 h). All the 60 animals were obtained from the Department of Physiology and Biophysics, Institute of Biomedical Sciences, University of Sao Paulo. Standard lab animal feed (52% carbohydrates, 21% proteins, and 4% lipids; Nuvilab CR1 (Nuvital, Brazil)) and water were provided *ad libitum*. All animals were diabetic and randomly assigned into two groups: diabetic without melatonin (non-supplemented group-NS) and diabetic treated with melatonin (supplemented group-M). The animals were then randomly subdivided into six groups with at least five animals per group: (I) non-supplemented non-exercised (NS 0 h); (II) non-supplemented euthanized immediately after exercise (NS IA); (III) non-supplemented euthanized 2 h after exercise (NS 2 h); (IV) melatonin-supplemented non-exercised (M 0 h); (V) melatonin-supplemented euthanized immediately after exercise (M IA); (VI) melatonin-supplemented euthanized 2 h after exercise (M 2 h).

Following a 1-week acclimatization period at our facility, experimental type 1 DM was induced by intraperitoneal injection of 70 mg/kg body weight (b.w.) of streptozotocin dissolved in citrate buffer (pH 4.2). The diabetic condition was confirmed 2 days after the streptozotocin injection by blood glucose levels greater than or equal to 200 mg/dL. Blood samples were drawn from cuts at the tip of the animals’ tails for the glucose estimation, using a glucose meter (Roche, Sao Paulo, Brazil).^20^

Melatonin was freshly prepared by dissolving in PBS (137 mM NaCl, 2.7 mM KCl, 8.1 mM Na_2_HPO_4_, 1.5 mM KH_2_PO_4_, pH 7.2-7.4, 0.2-µm filtered) and was administered intraperitoneally (20 mg per kg of body weight) once a day for 10 days. On the last day of treatment, the exhaustive exercise session was conducted 1 h before melatonin administration, as the half-life of melatonin is short (from 30 to 60 min). After this period, melatonin is excreted from the body.^21^ The non-supplemented animals received an identical volume of melatonin substituted with PBS as a control, *via* the same method of administration.

### Physical habituation to the exercise protocol

The exercise protocol was based on the model proposed by Leandro et al^22^ with minor modifications. On the 7th day after the induction of DM, the habituation process was carried out in a treadmill (Inbraport®) for 10 d before being subjected to the exhaustive session. This habituation regimen consisted of daily exercise practice for 15 min at a speed of 5 m/min. During exhaustive exercise, the treadmill was initially set at 5 m/min, and the speed was increased by 5 m/min at 3-min intervals until 16 m/min was reached. The treadmill was inclined by 20% for this exercise. Exhaustion was defined as when the rats lost motor control and showed a difficulty/delay to return to their normal position when placed on their backs.^23^ Animals were euthanized before (non-exercised group), immediately after the exhaustive exercise (IA group) and 2 h after the exhaustive exercise (2 h group). Animals’ hearts were collected and stored at −80 °C for further analysis. Tissues were frozen in liquid nitrogen and powdered by grinding using a mortar and pestle to ensure the best possible tissue homogeneity between assays. Additionally, a homogenizer (Ultra-Turrax - IKA, Staufen, Germany) was used to complete homogenize the heart tissue in the selected extraction buffers.

### Protein quantification

The determination of protein content was performed according to the method proposed by Bradford.^24^ Colorimetric reaction and absorbance were measured at 595 nm by a spectrophotometer (Molecular Devices – Espectra Max 190). The obtained absorbance results were used in the calculation of the equation of the line of a standard curve of Bovine Serum Albumin.

### Assessment of nitrite content

Analysis of nitrite content in hearts of animals was conducted through the previously proposed method^25^ in which nitrite reacts with sulfanilamide under acidic conditions and the reaction product reacts with N-chloridrate (l-naphtyl) ethylenediamine (NED) that generates a compound with an intense red color. Absorbance readings were performed by a spectrophotometer (Molecular Devices–Espectra Max 190).

### Determination of TBARS

For measurement of TBARS, the previously proposed method^26^ was applied. Concentrations of TBARS were then determined using the values obtained in a standard curve and reported as µM/mg of protein. The absorbance was measured at 532 nm in a spectrophotometer (Molecular Devices – Espectra Max 190).

### Reduced glutathione

Measurement of reduced glutathione was taken through its reaction with 5,5′-dithio-bis(2-nitrobenzoic acid) (DTNB), forming 5′-thio-2-nitrobenzoic acid (TNB), which can be measured at 414 nm by the spectrophotometer (Molecular Devices-Espectra Max 190).^27^ Results are presented in nmol per mg of protein.

### Western blotting for antioxidant enzyme expression

Animals’ hearts were homogenized in extraction buffer (100 mM Trizma pH 7.5, 10 mM EDTA, 100 mM NaF, 10 mM sodium phosphate, 10 mM sodium orthovanadate, 2 mM PMSF, 0.01 mg/ml aprotinin at 4 °C). Supernatants were resuspended in Laemmli buffer and samples (50 µg protein each) were subjected to electrophoresis and incubated with the specific, corresponding antibody according to the previously described method.^28^ Blots images were taken using a gel documentation system (ChemiDoc XRS+ system, Bio-Rad Laboratories), and the bands quantified using the Image Studio Lite v5.2 software (LI-COR Biosciences, Lincoln, NE, USA).

### Real-time RT-PCR

The mRNA expression of the main cardiac antioxidant enzymes (CAT, GPX, SOD1, and SOD2) was evaluated by real-time RT-PCR,^29^ using the Step-One Plus equipment (Applied Biosystems). The real-time RT-PCR assay was performed using the SsoAdvanced Universal SYBR Green Supermix (Bio-Rad). It was used 0.1 mM of each primer (sense and antisense). Sequences of primers were designed using information contained in the public database GeneBank of the National Center for Biotechnology Information. The quantification was performed using two housekeeping genes,^30^ and the mRNA expression calculated by the delta-delta Ct method.^31^

### Total oxidant status (TOS)

TOS of animals’ hearts were conducted following the previously proposed method.^32^ Hydrogen peroxide was used as a standard, and results were expressed in micromolar equivalent of hydrogen peroxide per liter (μmol H_2_O_2_ Eq/mg protein). Absorbance was measured at 560 nm by a spectrophotometer (Molecular Devices – Espectra Max 190).

### Total antioxidant status (TAS)

TAS measurement was performed using the previously developed method.^33^ This assay is based on the sample capacity against powerful ROS, such as hydroxyl (OH) radical. A known antioxidant (Trolox) was used as a standard and the results were expressed as mmol Trolox Eq/mg protein. Absorbance was measured at 444 nm by a spectrophotometer (Molecular Devices – Espectra Max 190).

### Oxidative stress index (OSI)

OSI was calculated by the ratio of TOS to TAS, according to the following formula: OSI (arbitrary unit) = TOS (μmol H_2_O_2_ Eq/mg protein) / TAS (mmol Trolox Eq/mg protein).^34^

### Statistical analysis

All data were presented as mean (M) and standard error of mean (SE). The exact values for M and SE are presented in Supplementary Table 1. Data were analyzed using Generalized Linear Models (GzLM) with Fisher`s post-hoc test. The significance level was set at 5%. Cohen’s *d* effect size was also calculated. We only considered as evidence data that presented large to huge effect sizes. The classification used was previously described as follows: small effect (≥0.20 to <0.49); medium effect (≥0.50 to <0.79); large effect (≥0.80).^35^ The software used was IBM® SPSS® Statistics v27 (IBM Corporation, Armonk, NY, USA).

## Results

### Oxidative stress biomarkers (Nitrite and TBARS levels) and total glutathione level

**Fig. 1:**
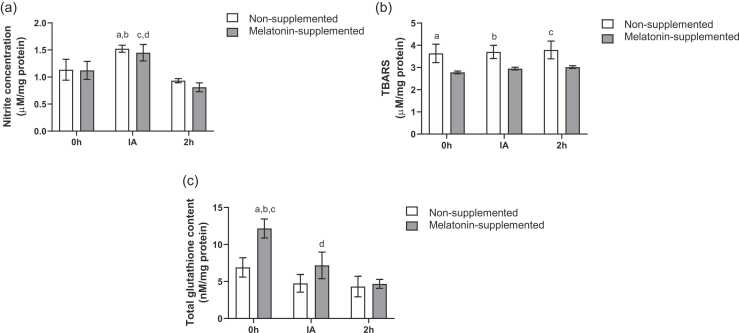
Protective effects of melatonin on oxidative stress biomarkers. (a) Effects of melatonin supplementation and time after the exhaustive exercise on nitrite concentration (µM/mg protein) of diabetic rats’ hearts. ^a^*P* = .02 *vs* Non-supplemented 0 h; ^b^*P* < .01 *vs* Non-supplemented 2 h; ^c^*P* = 0.05 *vs* Melatonin-supplemented 0 h; ^d^*P* < .01 *vs* Melatonin-supplemented 2 h; (b) Effects of melatonin supplementation and time after the exhaustive exercise on TBARS content (µM/mg protein) on diabetic rats’ hearts. ^a^*P* < .01 *vs* Melatonin-supplemented 0 h; ^b^*P* = .01 *vs* Melatonin-supplemented IA; ^c^*P* = .01 *vs* Melatonin-supplemented 2 h; (c) Effects of melatonin supplementation and time after the exhaustive exercise on total glutathione content (nM/mg protein) on diabetic rats’ hearts. ^a^*P* < .01 *vs* Non-supplemented 0 h; ^b^*P* < .01 *vs* Melatonin-supplemented IA; ^c^*P* < .01 *vs* Melatonin-supplemented 2 h; ^d^*P* = .146, *d* = 0.78 *vs* Non-supplemented IA. Results are presente^d^ as mean ± SEM (*n* = 5 for each group). 0 h = No exercise; IA = immediately after the exhaustive exercise; 2 h = 2 h after the exhaustive exercise.

As shown in Figure 1A, our analysis of nitrite levels revealed no significant differences between the non-supplemented and melatonin-supplemented groups at any of the evaluated time points. However, a significant time-dependent effect of exercise was observed within both groups (χ² = 26.92, *P* < .01). Specifically, a transient increase in nitrite was observed immediately after exercise (IA) when compared to the basal state (0 h) in both the non-supplemented (*P* = .02) and the melatonin-supplemented groups (*P* = .05).

In contrast, TBARS levels were not different based on the time of euthanasia, but showed a significant modulation due to supplementation (χ² = 22.36, *P* < .01) (Figure 1B). When comparing the non-supplemented and melatonin-supplemented groups, Fisher's post-hoc test revealed a significant difference at time 0 h (*P* < .01), immediately after exercise (*P* = .01), and 2 h after exercise (*P* = .01), with the melatonin-supplemented animals showing lower TBARS levels at all points.

Total glutathione presented significant differences that were dependent on both euthanasia time after exercise (χ² = 18.40, *P* < .01) and supplementation (χ² = 7.39, *P* < .01) (Figure 1C). The difference between groups occurred only at 0 h, where animals supplemented with melatonin presented 76% higher values than the non-supplemented group (*P* < .01). The effect size analysis also supports a difference between the melatonin-supplemented and non-supplemented groups at time IA (medium effect size-Cohen’s *d* = 0.78). With regards to time, the post-hoc test indicated that in melatonin-supplemented animals, the high values of glutathione observed at 0 h were significantly reduced when compared to the IA (*P* < .01) and 2 h (*P* < .01) groups.

### Antioxidant enzyme protein and mRNA expression

**Fig. 2:**
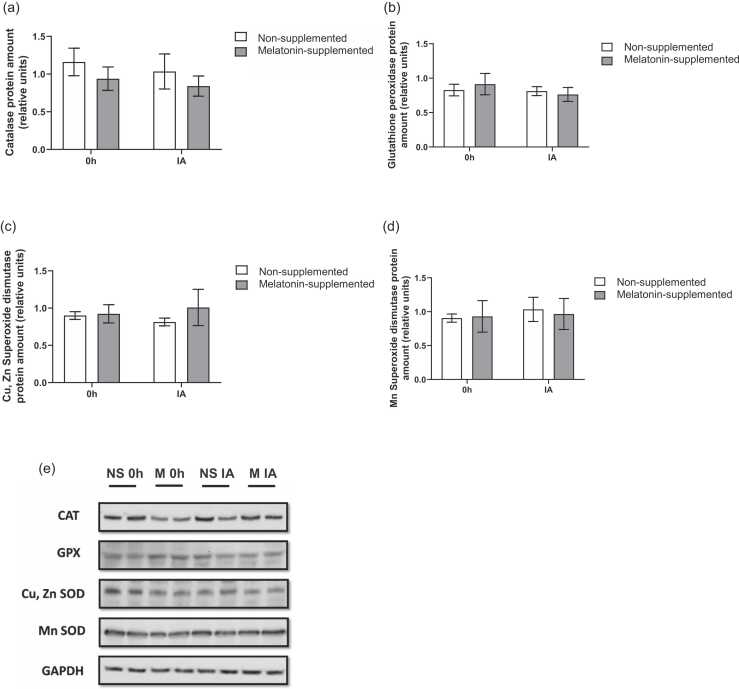
Melatonin modulation of antioxidant enzymes protein expression. (a) Effects of melatonin supplementation and time after the exhaustive exercise on catalase protein amount (relative units) of diabetic rats’ hearts; (b) Effects of melatonin supplementation and time after the exhaustive exercise on glutathione peroxidase protein amount (relative units) of diabetic rats’ hearts; (c) Effects of melatonin supplementation and time after the exhaustive exercise on Cu, Zn SOD1 protein amount (relative units) of diabetic rats’ hearts; (d) Effects of melatonin supplementation and time after the exhaustive exercise on Mn SOD2 protein amount (relative units) of diabetic rats’ heart. Results are presented as mean ± SEM (*n* = 5 for each group). 0 h = No exercise; IA = immediately after the exhaustive exercise; (e) Image of representative blots (in duplicates) of all investigated proteins. CAT = Catalase; GPX = Glutathione peroxidase; SOD = Superoxide dismutase; NS = Non-supplemented; M = Melatonin-supplemented; 0 h = No exercise; IA = immediately after the exhaustive exercise.

No differences were found when evaluating the protein expression of the selected antioxidant enzymes: catalase, GPx, SOD1, and SOD2 (Figure 2A-D, respectively).

**Fig. 3:**
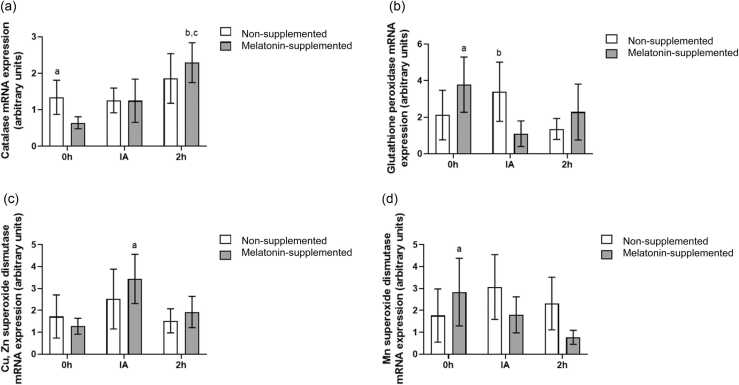
Melatonin modulation of antioxidant enzymes mRNA expression. (a) Effects of melatonin supplementation and time after the exhaustive exercise on catalase mRNA expression (arbitrary units) of diabetic rats’ hearts. ^a^*P* = .254, *d* = 0.89 *vs* Melatonin-supplemented 0 h; ^b^*P* = .108, *d* = 0.85 *vs* Melatonin-supplemented IA, ^c^*P* = .01 *vs* Melatonin-supplemented 2 h; (b) Effects of melatonin supplementation and time after the exhaustive exercise on glutathione peroxidase mRNA expression (arbitrary units) of diabetic rats’ hearts. ^a^*P* = .189, *d* = 1.02 *vs* Melatonin-supplemented IA; ^b^*P* = .215, *d* = 0.82 *vs* Melatonin-supplemented IA (c) Effects of melatonin supplementation and time after the exhaustive exercise on Cu, Zn SOD1 mRNA expression (arbitrary units) of diabetic rats’ hearts. ^a^*P* = 0.269, *d* = 1.15 *vs* Melatonin-supplemented 0 h; (d) Effects of melatonin supplementation and time after the exhaustive exercise on Mn SOD2 mRNA expression (arbitrary units) of diabetic rats’ heart. ^a^*P* = .222, *d* = 0.78 *vs* Melatonin-supplemented 2 h; Results are presented as mean ± SEM (*n* = 5 for each group). 0 h = No exercise; IA = immediately after the exhaustive exercise; 2 h = 2 h after the exhaustive exercise.

However, the analysis of mRNA expression revealed several modulations (Figure 3). Starting with catalase, the GzLM showed a significant difference only when considering the euthanasia time after exercise (χ² = 6.33, *P* = .04). According to the post-hoc test, melatonin-supplemented animals presented higher CAT mRNA expression at time 2 h compared to 0 h (*P* = .01). In addition, the effect size calculation showed a large effect difference in the melatonin-supplemented group at time 2 h compared to time IA (Cohens d = 0.85), and also when comparing non-supplemented and melatonin-supplemented groups at time 0 h (Cohens d = 0.89).

For GPx, the GzLM did not present differences for comparisons between groups or among times. However, our evaluation of effect sizes showed that in the melatonin-supplemented group, there was a lower value of GPX mRNA expression at time IA compared to 0 h (large effect size-Cohen’s *d* = 1.02). Furthermore, a large effect size (Cohen’s *d* = 0.82) was observed in the comparison between groups, where at time IA, the non-supplemented group presented higher GPX mRNA expression than the melatonin-supplemented group.

Both isoforms of superoxide dismutase (Cu, Zn-SOD and Mn-SOD) were not modulated by supplementation or euthanasia time according to GzLM results. Nevertheless, when we performed the analysis of effect sizes, we observed in the melatonin-supplemented group a higher mRNA expression of Cu, Zn-SOD at time IA compared to 0 h (large effect size-Cohen's *d* = 1.15), and a higher Mn-SOD mRNA expression at 0 h compared to 2 h (medium effect size-Cohen's *d* = 0.78).

### Oxidative stress status (TOS, TAS, and oxidative stress index)

**Fig. 4:**
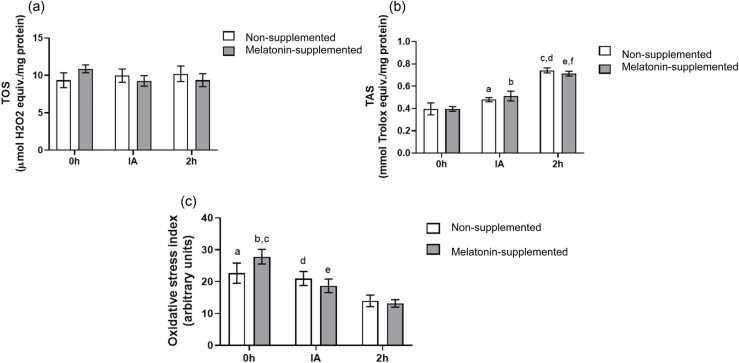
Melatonin effects on oxidative stress status. (a) Effects of melatonin supplementation and time after the exhaustive exercise on TOS (µmol H_2_O_2_ equiv./mg protein) of diabetic rats’ hearts; (b) Effects of melatonin supplementation and time after the exhaustive exercise on TAS (mmol Trolox equiv./mg protein) of diabetic rats’ heart. ^a^*P* = .04 *vs* Non-supplemented 0 h; ^b^*P* < .01 *vs* Melatonin-supplemented 0 h; ^c^*P* < .01 *vs* Non-supplemented 0 h; ^d^*P* < .01 *vs* Non-supplemented IA; ^e^*P* < .01 *vs* Melatonin-supplement^e^d 0 h; ^f^*P* < .01 *vs* Melatonin-supplemented IA; (C) Effects of melatonin supplementation and time after the exhaustive exercise on the OSI (arbitrary units) of diabetic rats’ heart. ^a^*P* < .01 *vs* Non-supplemented 2 h; ^b^*P* < .01 *vs* Melatonin-supplemented IA; ^c^*P* < .01 *vs* Melatonin-supplemented 2 h; ^d^*P* < .01 *vs* Non-supplemented 2 h; ^e^*P* = .01 *vs* Melatonin-supplemented 2 h. Results are presented as mean ± SEM (*n* = 5 for each group). 0 h = No exercise; IA = immediately after the exhaustive exercise; 2 h = 2 h after the exhaustive exercise.

As observed in Figure 4A, no differences were found for the TOS measurements. On the other hand, TAS showed a modulation dependent on the euthanasia time after exercise (χ² = 132.98, *P* < .01) (Figure 4B). For the non-supplemented group, Fisher’s post-hoc test indicated that TAS was higher 2 h after exercise when compared to 0 h (*P* < .01) and to the IA group (*P* < .01). The values of TAS in the IA group were also shown to be higher than the 0 h group (*P* = .04). Similar results were obtained in the melatonin-supplemented group, with differences among all time points, where the 2 h group presented the highest values compared to the IA group (*P* < .01) and the 0 h group (*P* < .01), and the IA group also showed higher values than the 0 h group (*P* < .01).

The oxidative stress index (OSI) also presented a difference that was dependent on euthanasia time after exercise (χ² = 38.56, *P* < .01) (Figure 4C). Fisher’s post-hoc test revealed that for the non-supplemented group, a lower OSI was found 2 h after exercise compared to time 0 h (*P* < .01) and IA (*P* < .01). Similar results were obtained in the melatonin-supplemented group, with lower values after 2 h of exercise compared to group 0 h (*P* < .01) and to group IA (*P* = .01). However, in the melatonin-supplemented group, a difference in OSI was also observed between times 0 h and IA (*P* < .01).

## Discussion

The findings of this study demonstrate that melatonin supplementation confers significant antioxidant protection to the diabetic heart, particularly when challenged by an acute session of exhaustive exercise. This primary contribution extends our understanding beyond melatonin's established effects under basal or chronic training conditions, highlighting its crucial role in enhancing cardiac resilience to a sudden, high-intensity stressor. Overall, our results support the original hypothesis that melatonin supplementation confers beneficial effects against oxidative stress induced by either diabetes or exhaustive exercise. By capturing these effects in a dynamic, time-resolved manner, our study provides novel insight into the immediate molecular preparations conferred by melatonin that allow the chronically diseased heart to better resist to an acute oxidative event.

The data is also in keeping with recent studies demonstrating the role of melatonin in cardiovascular health and disease. In several models of heart failure, melatonin was shown to be protective against damage induced by radiation, hyperthyroidism, chemotherapy, sepsis, and chronic Chagas disease.^36^ Additionally, melatonin can be beneficial in protecting against a number of risk factors that are present in diabetes, including diabetes-induced cardiomyopathy.^37^

The hyperglycemia observed in diabetes is principally responsible for the increase in reactive species production.^38^ One such species is nitric oxide (NO), a RNS produced by nitric oxide synthase (NOS). While important for vasodilation, NO is often downregulated in diabetes.^39^ In the present study, we evaluated nitrite content and found a significant modulation only by exercise. In both the non-supplemented and supplemented groups, values obtained immediately after exercise were higher than at other time points, consistent with an increase in NO induced by the exercise session.^40^ The role of melatonin in modulating NO is complex and context-dependent. While melatonin has been shown to control excessive NO production driven by pathological inducible NOS (iNOS)^41^, ^42^, the increase in nitrite observed in our model is likely a physiological, eNOS-mediated response essential for coronary vasodilation during exertion.^43^ In this context, the powerful stimulus of exhaustive exercise was the dominant regulator of NO production, a physiological process with which melatonin did not interfere.

Besides nitrite, we also evaluated lipid peroxidation *via* TBARS. In contrast to nitrite, TBARS levels were significantly modulated by supplementation at all time points. The consistent and robust reduction in TBARS by melatonin can be explained by its powerful dual antioxidant mechanism. Firstly, as a direct scavenger, melatonin neutralizes the hydroxyl radicals that initiate the lipid peroxidation cascade.^13^ Secondly, melatonin provides indirect protection by stimulating the synthesis of other major antioxidants. This is strongly supported by our findings on glutathione, a major non-enzymatic antioxidant. The melatonin-supplemented group demonstrated higher basal glutathione levels, which is consistent with reports that melatonin upregulates GSH synthesis.^44^ This molecule is considered a major cellular antioxidant capable of efficiently inhibiting ROS damages and eliminating products of lipid peroxidation.^45^, ^46^ The decrease on glutathione levels observed at times IA and 2 h in the melatonin-supplemented group, compared to 0 h, suggests that rats supplemented with melatonin presented a better antioxidant dynamic, which was required immediately after the demand of exercise.

Regarding the enzymatic antioxidant system, our results showed no modulation in the protein levels of any of the enzymes. As a matter of fact, there is conflicting data in the literature regarding the relationship of antioxidant enzymes levels and diabetes.^47^, ^48^, ^49^ An intriguing finding of our study was the modulation of antioxidant enzyme mRNA levels (e.g., CAT, GPX) without corresponding changes in their total protein content. This discrepancy suggests that the regulation of the cardiac antioxidant system in this model may occur at a post-transcriptional level. This finding of an early transcriptional response contrasts with studies employing chronic interventions. For instance, Rahbarghazi et al^19^ observed increased SOD and GPx enzyme activity in diabetic mice after four weeks of exercise training. This suggests that while gene transcription is rapidly initiated in response to an acute stressor, significant alterations to the total protein pool likely require a more prolonged or repeated stimulus. Factors such as mRNA stability, translation efficiency, or protein degradation rates, which were not measured in this study, could be playing a more significant role than transcription alone. For instance, melatonin, a well-known pleiotropic regulator, has been reported to influence protein turnover and mRNA stability, which could explain these observations.^41^, ^50^

This transcriptional response may be linked to the Nrf2/ARE pathway, a major mechanism modulating cardiac defense, which is often depressed in diabetic hearts^51^, ^52^ but can be upregulated by melatonin.^53^ The lower antioxidant gene expression of CAT and GPX on supplemented animals at rest may be explained by the increase on non-enzymatic antioxidant compounds, as we observed in GSH content. The acute exercise was sufficient to positively modulate these enzymes' mRNA expressions, which made us suggest that a chronic practice of exercise (exercise training) would be essential to fully enhance the enzymatic antioxidant system.

Finally, after evaluation of TOS, TAS, and their ratio (OSI), we found that these parameters were influenced mainly by the time of exercise. Both groups presented a suitable response for the physiological demand induced by the exercise through increasing their antioxidant capacity. This positive adaptation may explain why TOS values were not modulated. While these assays may suggest that melatonin was not efficient to modulate the overall oxidative stress, it is important to highlight that they may not be sensitive enough to detect specific types of reactive species. The fact that TBARS levels were shown to be lower in the melatonin-supplemented group provides more direct evidence of protection, a finding also observed in a recent study with diabetic patients.^54^

## Conclusion

Taken together, the results of this study suggest that melatonin provides antioxidant protection to the heart of diabetic animals and that this response can be further intensified by exercise. Melatonin was shown to control lipid peroxidation and increase glutathione levels, demonstrating its potential as a therapeutic supplement for diabetic patients. Our findings indicate that the modulation of the antioxidant system by melatonin occurs in a time-dependent manner, beginning with non-enzymatic defenses. While a longer supplementation period or the implementation of regular exercise training may be required to induce further improvements in the cardiac antioxidant system, our study provides a key contribution to the existing knowledge by specifically highlighting melatonin's role in enhancing the cardiac resilience of the diabetic heart against acute, ROS-induced damage.

## CRediT authorship contribution statement

**Carol Nguyen:** Writing – review & editing, Writing – original draft, Methodology, Investigation, Formal analysis. **Rafael Ishihara Figueiroa:** Writing – review & editing, Writing – original draft, Methodology, Formal analysis. **Cristiano Mendes da Silva:** Writing – review & editing, Resources, Funding acquisition. **Elaine Hatanaka:** Writing – review & editing, Methodology, Conceptualization. **Gary Sweeney:** Writing – review & editing, Supervision, Methodology, Conceptualization. **Rafael Herling Lambertucci:** Writing – review & editing, Writing – original draft, Methodology, Supervision, Project administration, Investigation, Formal analysis, Conceptualization, Funding acquisition.

## Declaration of generative AI and AI-assisted technologies in the writing process

During the preparation of this work, the author(s) used Gemini in order to improve language and readability. After using this tool/service, the author(s) reviewed and edited the content as needed and take(s) full responsibility for the content of the publication.

## Declaration of Interest

The authors declare that they have no known competing financial interests or personal relationships that could have appeared to influence the work reported in this paper.

## Data Availability

Data will be made available on request.

## References

[1] Toniolo A., Cassani G., Puggioni A. (2019). The diabetes pandemic and associated infections: suggestions for clinical microbiology. Rev Med Microbiol.

[2] Cho N.H., Shaw J.E., Karuranga S. (2018). IDF diabetes atlas: global estimates of diabetes prevalence for 2017 and projections for 2045. Diabetes Res Clin Pract.

[3] Chamine I., Larson A., Huguet N. (2022). Characterizing acute and chronic complications among patients with diabetes mellitus in community health centers. Ann Fam Med.

[4] Faselis C., Katsimardou A., Imprialos K., Deligkaris P., Kallistratos M., Dimitriadis K. (2020). Microvascular complications of type 2 diabetes mellitus. Curr Vasc Pharmacol.

[5] De Geest B., Mishra M. (2022). Role of oxidative stress in diabetic cardiomyopathy. Antioxidants.

[6] Peng M.L., Fu Y., Wu C.W., Zhang Y., Ren H., Zhou S.S. (2022). Signaling pathways related to oxidative stress in diabetic cardiomyopathy. Front Endocrinol (Lausanne).

[7] Byrne N.J., Rajasekaran N.S., Abel E.D., Bugger H. (2021). Therapeutic potential of targeting oxidative stress in diabetic cardiomyopathy. Free Radic Biol Med.

[8] Zhang Z., Huang Q., Zhao D., Lian F., Li X., Qi W. (2023). The impact of oxidative stress-induced mitochondrial dysfunction on diabetic microvascular complications. Front Endocrinol (Lausanne).

[9] Pisoschi A.M., Pop A. (2015). The role of antioxidants in the chemistry of oxidative stress: a review. Eur J Med Chem.

[10] Matough F.A., Budin S.B., Hamid Z.A., Alwahaibi N., Mohamed J. (2012). The role of oxidative stress and antioxidants in diabetic complications. Sultan Qaboos Univ Med J.

[11] Seifried H.E., Anderson D.E., Fisher E.I., Milner J.A. (2007). A review of the interaction among dietary antioxidants and reactive oxygen species. J Nutr Biochem.

[12] Vazquez J., Gonzalez B., Sempere V., Mas A., Torija M.J., Beltran G. (2017). Melatonin reduces oxidative stress damage induced by hydrogen peroxide in saccharomyces cerevisiae. Front Microbiol.

[13] Reiter R.J., Mayo J.C., Tan D.X., Sainz R.M., Alatorre-Jimenez M., Qin L. (2016). Melatonin as an antioxidant: under promises but over delivers. J Pineal Res.

[14] Monteiro K., Shiroma M.E., Damous L.L. (2024). Antioxidant actions of melatonin: a systematic review of animal studies. Antioxidants (Basel).

[15] Emamgholipour S., Hossein-Nezhad A., Ansari M. (2016). Can melatonin act as an antioxidant in hydrogen peroxide-induced oxidative stress model in human peripheral blood mononuclear cells?. Biochem Res Int.

[16] Fischer T.W., Kleszczynski K., Hardkop L.H., Kruse N., Zillikens D. (2013). Melatonin enhances antioxidative enzyme gene expression (CAT, GPx, SOD), prevents their UVR-induced depletion, and protects against the formation of DNA damage (8-hydroxy-2′-deoxyguanosine) in ex vivo human skin. J Pineal Res.

[17] Chitimus D.M., Popescu M.R., Voiculescu S.E. (2020). Melatonin's impact on antioxidative and anti-inflammatory reprogramming in homeostasis and disease. Biomolecules.

[18] Socaciu A.I., Ionut R., Socaciu M.A. (2020). Melatonin, an ubiquitous metabolic regulator: functions, mechanisms and effects on circadian disruption and degenerative diseases. Rev Endocr Metab Disord.

[19] Rahbarghazi A., Siahkouhian M., Rahbarghazi R. (2021). Melatonin and prolonged physical activity attenuated the detrimental effects of diabetic condition on murine cardiac tissue. Tissue Cell.

[20] Alba-Loureiro T.C., Pithon-Curi T.C., Curi R. (2008). Reduced cytokine production by glycogen-elicited peritoneal cells from diabetic rats. Shock.

[21] Mallo C., Zaidan R., Galy G. (1990). Pharmacokinetics of melatonin in man after intravenous infusion and bolus injection. Eur J Clin Pharmacol.

[22] Leandro C.G., Levada A.C., Hirabara S.M. (2007). A program of moderate physical training for Wistar rats based on maximal oxygen consumption. J Strength Cond Res.

[23] Silva E.P., Borges L.S., Mendes-da-Silva C., Hirabara S.M., Lambertucci R.H. (2017). l-Arginine supplementation improves rats' antioxidant system and exercise performance. Free Radic Res.

[24] Bradford M.M. (1976). A rapid and sensitive method for the quantitation of microgram quantities of protein utilizing the principle of protein-dye binding. Anal Biochem.

[25] Ding A.H., Nathan C.F., Stuehr D.J. (1988). Release of reactive nitrogen intermediates and reactive oxygen intermediates from mouse peritoneal macrophages. Comparison of activating cytokines and evidence for independent production. J Immunol.

[26] Janero D.R. (1990). Malondialdehyde and thiobarbituric acid-reactivity as diagnostic indices of lipid peroxidation and peroxidative tissue injury. Free Radic Biol Med.

[27] Tietze F. (1969). Enzymic method for quantitative determination of nanogram amounts of total and oxidized glutathione: applications to mammalian blood and other tissues. Anal Biochem.

[28] Towbin H., Staehelin T., Gordon J. (1979). Electrophoretic transfer of proteins from polyacrylamide gels to nitrocellulose sheets: procedure and some applications. Proc Natl Acad Sci U S A.

[29] Higuchi R., Dollinger G., Walsh P.S., Griffith R. (1992). Simultaneous amplification and detection of specific DNA sequences. Biotechnology (N Y).

[30] Vandesompele J., De Preter K., Pattyn F. (2002). Accurate normalization of real-time quantitative RT-PCR data by geometric averaging of multiple internal control genes. Genome Biol.

[31] Hellemans J., Mortier G., De Paepe A., Speleman F., Vandesompele J. (2007). qBase relative quantification framework and software for management and automated analysis of real-time quantitative PCR data. Genome Biol.

[32] Erel O. (2005). A new automated colorimetric method for measuring total oxidant status. Clin Biochem.

[33] Erel O. (2004). A novel automated direct measurement method for total antioxidant capacity using a new generation, more stable ABTS radical cation. Clin Biochem.

[34] Cingi Yirun M., Unal K., Altunsoy Sen N., Yirun O., Aydemir C., Goka E. (2016). Evaluation of oxidative stress in bipolar disorder in terms of total oxidant status, total antioxidant status, and oxidative stress index. Noro Psikiyatr Ars.

[35] Sawilowsky S. (2009). New effect size rules of thumb. J Modern Appl Stat Methods.

[36] Nduhirabandi F., Maarman G.J. (2018). Melatonin in heart failure: a promising therapeutic strategy?. Molecules.

[37] Amin A.H., El-Missiry M.A., Othman A.I. (2015). Melatonin ameliorates metabolic risk factors, modulates apoptotic proteins, and protects the rat heart against diabetes-induced apoptosis. Eur J Pharmacol.

[38] Afanas'ev I. (2010). Signaling of reactive oxygen and nitrogen species in Diabetes mellitus. Oxid Med Cell Longev.

[39] Noyman I., Marikovsky M., Sasson S. (2002). Hyperglycemia reduces nitric oxide synthase and glycogen synthase activity in endothelial cells. Nitric Oxide.

[40] Tsukiyama Y., Ito T., Nagaoka K., Eguchi E., Ogino K. (2017). Effects of exercise training on nitric oxide, blood pressure and antioxidant enzymes. J Clin Biochem Nutr.

[41] Hardeland R., Cardinali D.P., Srinivasan V., Spence D.W., Brown G.M., Pandi-Perumal S.R. (2011). Melatonin--a pleiotropic, orchestrating regulator molecule. Prog Neurobiol.

[42] Akbulut K.G., Guney S., Cetin F., Akgun H.N., Aktas S.H., Akbulut H. (2013). Melatonin delays brain aging by decreasing the nitric oxide level. Neurophysiology.

[43] Gielen S., Schuler G., Adams V. (2010). Cardiovascular effects of exercise training: molecular mechanisms. Circulation.

[44] Winiarska K., Fraczyk T., Malinska D., Drozak J., Bryla J. (2006). Melatonin attenuates diabetes-induced oxidative stress in rabbits. J Pineal Res.

[45] Qin T., Feng D., Zhou B., Bai L., Yin Y. (2022). Melatonin suppresses LPS-induced oxidative stress in dendritic cells for inflammatory regulation via the Nrf2/HO-1 axis. Antioxidants (Basel).

[46] Das N., Mandala A., Naaz S. (2017). Melatonin protects against lipid-induced mitochondrial dysfunction in hepatocytes and inhibits stellate cell activation during hepatic fibrosis in mice. J Pineal Res.

[47] Salmanoglu D.S., Gurpinar T., Vural K., Ekerbicer N., Dariverenli E., Var A. (2016). Melatonin and L-carnitin improves endothelial disfunction and oxidative stress in Type 2 diabetic rats. Redox Biol.

[48] Rodriguez C., Mayo J.C., Sainz R.M. (2004). Regulation of antioxidant enzymes: a significant role for melatonin. J Pineal Res.

[49] Song C., Peng W., Yin S. (2016). Melatonin improves age-induced fertility decline and attenuates ovarian mitochondrial oxidative stress in mice. Sci Rep.

[50] Mayo J.C., Sainz R.M., Antoli I., Herrera F., Martin V., Rodriguez C. (2002). Melatonin regulation of antioxidant enzyme gene expression. Cell Mol Life Sci.

[51] Ge Z.D., Lian Q., Mao X., Xia Z. (2019). Current status and challenges of NRF2 as a potential therapeutic target for diabetic cardiomyopathy. Int Heart J.

[52] Tan Y., Ichikawa T., Li J. (2011). Diabetic downregulation of Nrf2 activity via ERK contributes to oxidative stress-induced insulin resistance in cardiac cells in vitro and in vivo. Diabetes.

[53] Xu C., Wang J., Fan Z. (2021). Cardioprotective effects of melatonin against myocardial ischaemia/reperfusion injury: activation of AMPK/Nrf2 pathway. J Cell Mol Med.

[54] Raygan F., Ostadmohammadi V., Bahmani F., Reiter R.J., Asemi Z. (2019). Melatonin administration lowers biomarkers of oxidative stress and cardio-metabolic risk in type 2 diabetic patients with coronary heart disease: a randomized, double-blind, placebo-controlled trial. Clin Nutr.

