# Supplementary material for: Melatonin attenuates cardiac oxidative stress in diabetic rats following acute exhaustive exercise
Source: Cell Stress Chaperones. 2025 Oct 17;30(6):100126. doi: 10.1016/j.cstres.2025.100126 (PMC12616075; doi:10.1016/j.cstres.2025.100126)
Supplement: Supplementary file 1 — Supplementary material [file mmc1.pdf]

Supplementary Table 1

Oxidative stress biomarkers

	Time	Condition	Mean	SE
Nitrite concentration (µM/mg protein)	0h	Diabetic	1.134	0.1926
		Diabetic + Melatonin	1.123	0.1668
	IA	Diabetic	1.523	0.0656
		Diabetic + Melatonin	1.449	0.1515
	2h	Diabetic	0.933	0.0380
		Diabetic + Melatonin	0.810	0.0817
TBARS content (µM/mg protein)	0h	Diabetic	3.635	0.4148
		Diabetic + Melatonin	2.780	0.0605
	IA	Diabetic	3.701	0.2980
		Diabetic + Melatonin	2.949	0.0590
	2h	Diabetic	3.791	0.3998
		Diabetic + Melatonin	3.016	0.0599
Total glutathione content (nM/mg protein)	0h	Diabetic	6.900	13.036
		Diabetic + Melatonin	12.164	12.841
	IA	Diabetic	4.750	11.909
		Diabetic + Melatonin	7.178	17.964
	2h	Diabetic	4.324	13.877
		Diabetic + Melatonin	4.670	0.6057

Antioxidant enzymes protein expression

	Time	Condition	Mean	SE
Catalase protein amount (relative units)	0h	Diabetic	1.161	0.1826
		Diabetic + Melatonin	0.939	0.1550
	IA	Diabetic	1.035	0.2323
		Diabetic + Melatonin	0.841	0.1341
Glutathione peroxidase protein amount (relative units)	0h	Diabetic	0.827	0.0847
		Diabetic + Melatonin	0.914	0.1552
	IA	Diabetic	0.812	0.0652
		Diabetic + Melatonin	0.763	0.1017
Cu, Zn SOD1 protein amount (relative units)	0h	Diabetic	0.900	0.0519
		Diabetic + Melatonin	0.923	0.1228
	IA	Diabetic	0.814	0.0515
		Diabetic + Melatonin	1.009	0.2433
Mn SOD2 protein amount (relative units)	0h	Diabetic	0.905	0.0612
		Diabetic + Melatonin	0.931	0.2323
	IA	Diabetic	1.034	0.1782
		Diabetic + Melatonin	0.966	0.2288

Antioxiadant enzymes mRNA expression

	Time	Condition	Mean	SE
Catalase mRNA expression (arbitrary units)	0h	Diabetic	1340	0.470
		Diabetic + Melatonin	0.642	0.163
	IA	Diabetic	1254	0.340
		Diabetic + Melatonin	1247	0.593
	2h	Diabetic	1858	0.681
		Diabetic + Melatonin	2291	0.546
Glutathione peroxidase mRNA expression (arbitrary units)	0h	Diabetic	2121	1357
		Diabetic + Melatonin	3.785	1512
	IA	Diabetic	3397	1622
		Diabetic + Melatonin	1096	0.700
	2h	Diabetic	1.357	0.573
		Diabetic + Melatonin	2285	1525
Cu, Zn SOD1 mRNA expression (arbitrary units)	0h	Diabetic	1720	0.986
		Diabetic + Melatonin	1276	0.365
	IA	Diabetic	2518	1366
		Diabetic + Melatonin	3439	1128
	2h	Diabetic	1522	0.549
		Diabetic + Melatonin	1929	0.714
Mn SOD2 mRNA expression (arbitrary units)	0h	Diabetic	1764	1217
		Diabetic + Melatonin	2.835	1548
	IA	Diabetic	3070	1480
		Diabetic + Melatonin	1796	0.824
	2h	Diabetic	2.315	1203
		Diabetic + Melatonin	0.769	0.320

Oxidative stress status

	Time	Condition	Mean	SE
TAS (mmol Trolox equiv./mg protein)	0h	Diabetic	0.396	0.0533
		Diabetic + Melatonin	0.396	0.0214
	IA	Diabetic	0.480	0.0188
		Diabetic + Melatonin	0.512	0.0439
	2h	Diabetic	0.742	0.0220
		Diabetic + Melatonin	0.713	0.0211
TOS (µmol H2O2 equiv./mg protein)	0h	Diabetic	9.356	0.9866
		Diabetic + Melatonin	10.864	0.5243
	IA	Diabetic	9.963	0.8868
		Diabetic + Melatonin	9.266	0.7000
	2h	Diabetic	10.208	10.314
		Diabetic + Melatonin	9.359	0.8716
OSI (arbitrary units)	0h	Diabetic	22.649	31.707
		Diabetic + Melatonin	27.824	23.036
	IA	Diabetic	20.972	22.045
		Diabetic + Melatonin	18.685	21.313
	2h	Diabetic	13.966	18.015
		Diabetic + Melatonin	13.140	11.618
